# Announcements

**Published:** 2015-04-24

## Air Quality Awareness Week — April 27–May 1, 2015

CDC is collaborating with the U.S. Environmental Protection Agency (EPA) to urge persons to learn how air quality affects health during Air Quality Awareness Week, April 27–May 1, 2015.

Although outdoor air quality has improved since the 1990s, many challenges remain. Ground-level ozone, the primary component of smog, and particle pollution are just two of the many factors that decrease air quality and might affect health. Particle pollution can cause eye, lung, and throat irritation and can cause a heart attack among persons with heart disease (1). Ozone exposure can worsen symptoms of asthma, bronchitis, or emphysema and can cause coughing and pain when taking a deep breath, lung and throat irritation, and wheezing and trouble breathing during exercise or outdoor activities (2).

EPA’s Air Quality Index (AQI) (3) predicts the level of pollution in the air each day and provides advice on healthy physical activity. The AQI is available on the internet, on many local TV weather forecasts, or as free e-mail tools and apps (4). The AQI includes information about the five major air pollutants in the United States that are regulated by EPA, including ozone and particle pollution.

Join experts from CDC, EPA, the National Oceanic and Atmospheric Administration, and the National Park Service on Thursday, April 30, at 1:00 pm Eastern for a TwitterChat about air quality, physical activity, and health. Use the hashtag *#AirQualityChat* in chat messages to join the conversation.

Additional air quality and health information is available at http://www.cdc.gov/air/default.htm and http://www.epa.gov/airnow/airaware.
